# RNA Interference Targeting *Snail* Inhibits the Transforming Growth Factor **β**2-Induced Epithelial-Mesenchymal Transition in Human Lens Epithelial Cells

**DOI:** 10.1155/2013/869101

**Published:** 2013-09-14

**Authors:** Ping Li, Jiaona Jing, Jianyan Hu, Tiejun Li, Yuncheng Sun, Huaijin Guan

**Affiliations:** ^1^Department of Ophthalmology, Affiliated Hospital of Nantong University, 20 Xisi Road, Nantong, Jiangsu 226001, China; ^2^Department of Ophthalmology, Yixing Hospital of Traditional Chinese Medicine, 128 Yangquan East Road, Yixing, Wuxi, Jiangsu 214200, China; ^3^Department of Life Science Center, Biomics Biotechnologies Co. Ltd., 76 Changxing Road, E&T Development Area, Nantong, Jiangsu 226016, China; ^4^Small RNA Technology and Application Institute, Nantong University, 76 Changxing Road, E&T Development Area, Nantong, Jiangsu 226016, China

## Abstract

Epithelial-msenchymal transition (EMT) contributes to posterior capsule opacification (PCO) type of cataract. Transcription factors *Snail* is a key trigger of EMT activated by transforming growth factor **β** (TGF**β**). This study was done to investigate the effect of *Snail* targeting siRNA on TGF**β**2-induced EMT in human lens epithelial cells. TGF**β**2 treatment of cultured human epithelial cell line (HLEB3) upregulated the expression of *Snail* and the EMT relevant molecules such as vimentin and **α**-SMA but downregulated the expression of keratin and E-cadherin. After the stimulation of TGF**β**2, the HLEB3 cells became fibroblast-like in morphology, and the junctions of cell-cell disappeared. TGF**β**2 treatment also enhanced migration ability of HLEB3 cells. TGF**β**2-induced *Snail* expression and EMT were significantly inhibited by *Snail* siRNA. By analyzing the response characteristics of HLEB3 in TGF**β**2-induced EMT model with/without *Snail*-specific siRNA, we concluded that *Snail* is an element in the EMT of HLEB3 cells induced by TGF**β**2. *Snail* siRNA targeting can block the induced EMT and therefore has the potential to suppress the development of PCO.

## 1. Introduction

Epithelial-mesenchymal transition (EMT) is a programmed development of biological cells characterized by loss of cell adhesion, repression of E-cadherin expression, increased cell mobility, and change of morphology. EMT is a highly conserved and fundamental process not only in development, but also in fibrosis, metastasis of tumor cells, and wound healing [1–4]. In cataract surgery, where entire lens content is removed, lens epithelial cells (LECs) can undergo EMT, migrate to the posterior capsular surface, and result in fibrosis of the posterior capsule as well as the residual anterior capsule [4–6]. Clinically, the EMT of LECs after cataract lens removal usually results in secondary cataract that can present as anterior polar cataracts and/or posterior capsular opacification [7, 8].

During EMT, epithelium cells undergo transdifferentiation toward a myofibroblastic phenotype. The two cell types have different skeletal proteins, keratin for epithelium and vimentin for myofibroblastic. The cells derived from surface ectoderm always express E-cadherin to form adherence to each other. The EMT process involves transcriptional reprogramming of a series of genes that include *α*-SMA known as a maker of myofibroblast cells. Therefore, except for the distinct expression of keratin and vimentin, the *α*-SMA expression is considered as the feature of LECs transdifferentiation as well as the loss of E-cadherin production [9–11]. 

TGF*β* is composed of homodimeric polypeptides that regulate many aspects of cellular function, including cell growth, differentiation, inflammation, and wound healing [12–14]. Numerous *in vitro* and *in vivo* studies have indicated the role of active TGF*β* in promoting an aberrant differentiation pathway and EMT of various epithelial tissues [15, 16]. Although five members of the TGF*β* family have currently been identified, only TGF*β* isoforms 1, 2, and 3 have been detected in mammals [17]. TGF*β*1 and TGF*β*2 are expressed in human lens and release abundantly in the ocular media [18]. The predominant form of TGF*β*1 and TGF*β*2 is in the latent [19] but can be activated under pathological conditions such as inflammation, fibrosis, trauma, and surgery after a 25-kDa dimer cleaved from its latent precursor [20]. The amount of TGF*β* in aqueous humor after cataract surgery with intraocular lens implantation ranged from 2.3 to 8.1 ng/mL with 61% of it present in the active form [21]. Normally, the activity of TGF*β* in the eye appears to be highly regulated by vitreous containing molecules [8]. TGF*β*2 is expressed at much higher levels than the other isoforms in the aqueous humor and vitreous and thus is likely to be a major mediator of EMT in LECs *in vivo* [22, 23].

The *Snail* family members are a group of transcription factors that are involved in regulation of EMT induced by TGF*β* during embryonic development and tumor progression [24–28]. They are involved in many embryonic processes, such as the ingression of the early mesodermal cells at gastrulation and the delamination of the neural crest from the neural tube [29]. In adult, *Snail* was mainly expressed in heart, lung, brain, and skeletal muscle, but there is no expression in most normal organs/tissues including eyes [30]. However, *Snail* can be expressed in ocular tissue under pathological conditions especially fibrotic diseases such as corneal scarring [31], subcapsular cataract [32], and proliferative vitreoretinopathy (PVR) [33]. Indeed, *Snail* is activated to induce EMT in mammalian cells and suppress the expression of E-cadherin [8, 34, 35]. Cho et al. have reported the role of *Snail* in ETM of mouse lens epithelial cells [36].

In the present study, we sought to confirm the involvement of *Snail *gene in TGF*β*2-induced EMT of human LECs and to test a novel hypothesis that the inhibition of *Snail* expression by siRNA can block TGF*β*2-induced EMT.

## 2. Material and Methods

### 2.1. Cells and Cell Culture

Human lens epithelial cell line HLEB3 was purchased from ATCC. Cells were cultured in Dulbecco's modified Eagle's medium (DMEM; Invitrogen, CA, USA) supplemented with 15% fetal bovine serum (FBS; Invitrogen, CA, USA). All culture medium contained no antibiotics. The TGF*β*2 treatment was carried out after the cells were incubated in serum-free medium for 24 hours, and 10 ng/mL of TGF*β*2 was added to the culture medium for the indicated times.

### 2.2. Reagents and Antibodies

Recombinant human TGF*β*2 was purchased from Peprotech (Rocky, Hill, NJ, USA). Anti-E-cadherin and keratin antibodies were purchased from Cell Signaling (Beverly, MA, USA). Anti-*Snail* and vimentin antibodies were obtained from Santa Cruz Biotechnology (Santa Cruz, CA, USA). Anti-*α*-SMA antibody was purchased from Abcam (Cambridge, MA, USA). CY3/FITC tagged secondary antibodies were from BOSTER (Wuhan, China).

### 2.3. siRNA and Transfection

According to Elbashir's principle [37], four siRNAs (P1–P4) targeting human *Snail* and one negative control siRNA (P5) were designed using web-based software (http://www.ambion.com/techlib/misc/siRNA_finder.html) and synthesized chemically (Biomics, Nantong, China) (Table 1). The siRNAs were transfected into HLEB3 cells by liposome Lipofectamine 2000 according to the manufacturer's protocol (Invitrogen, CA, USA). The siRNA treatment was performed before the TGF*β*2 stimulation. 

### 2.4. Quantification of *Snail* mRNA

Total RNA of HLEB3 cells was extracted for cDNA synthesis using RISO reagent (RISO; Biomics, Nantong, China). cDNA was synthesized by MLV reverse transcriptase using 2 *μ*g total RNA in a total volume of 20 *μ*L (QuantiTect, Qiagen, Germany). The *Snail* transcript was detected by quantitative RT-PCR using iCycler iQ System (Bio-Rad Laboratories, Hercules, CA, USA) and SYBR Green QPCR Master Mix (Biomics, Nantong, China). The primers for *snail* are forward 5′-TCGTCCTTCTCCTCTACTTCAG-3′ and reverse 5′-CGTGTGGCTTCGGATGTG-3′, which amplify a 201 bp target. For the internal control, GAPDH was amplified using primers forward 5′-GAAGGTGAAGGTCGGAGTC-3′ and reverse 5′-GAAGATGGTGATGGGATTTC-3′, which amplify a 226 bp target. Following PCR, a thermal melt profile was performed for amplicon identification. The specificity of the amplification reactions was also confirmed by agarose gel electrophoresis. The relative expression was presented as fold changes after normalizing to the GAPDH control.

### 2.5. Immunofluorescent Staining

HLEB3 cells were grown on glass coverslips before siRNAs were transfected and then exposed to 10 ng/mL of TGF*β*2 for 1 hour. Cells were fixed with 4% paraformaldehyde for 30 min at 4°C, followed by incubation with 0.1% Triton X-100 and 3% BSA for 2 h in room temperature for permeabilization and blocking. The primary antibodies (1 : 100) against Snail, vimentin, E-cadherin, keratin, or *α*-SMA diluted in PBS were placed on cells for overnight at 4°C, respectively, followed by incubation with CY3-conjugated goat anti-rabbit or FITC-conjugated goat anti-mouse immunoglobulin (1 : 200) for 2 hours at 37°C in the dark. The nuclei were counterstained with Hoechst 33258 (Invitrogen, CA, USA). Images were acquired with a fluorescence microscope (DM4000B, Leica, Germany).

### 2.6. Transwell Assay

Transwell apparatus with 8 *μ*m pore size membrane (Costar, Cambridge, MA, USA) was used to detect the migration ability of HLEB3 cells. The siRNAs-treated HLEB3 cells were exposed to 10 ng/mL of TGF*β*2 for 48 h. Serum-free DMEM containing 1 × 10^5^ cells in 100 *μ*L was added into the upper chamber; the lower chamber contained 500 *μ*L of 15% FBS-containing medium. After incubation at 37°C for 24 h, membranes were swabbed with a cotton swab, soaked in 0.1% crystal violet for 10 min, and then washed with PBS. The number of cells attached to the lower surface of the polycarbonate filter was counted at 100x magnification under a light microscope. 

### 2.7. Statistical Analysis

All results are expressed as the mean ± SD. The data were analyzed with ANOVA and SNK-q test using SPSS17.0. *P* < 0.05 was considered to be statistically significant.

## 3. Results

### 3.1. Expression of *Snail* Induced by TGF*β*2

**Figure 1:**
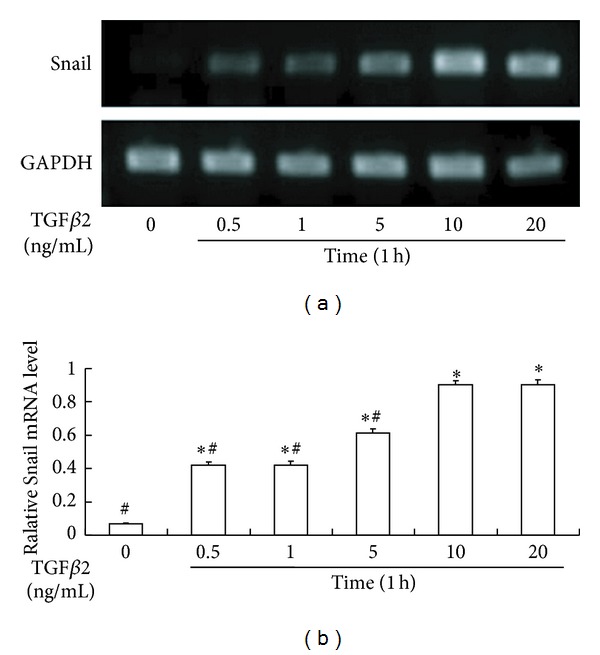
TGF*β*2-induced expression of Snail mRNA in a dose-dependent manner. (a) Representative agarose gel electrophoresis images of Snail and house gene expression after TGF*β*2 treatment. (b) The summary of triplicated experiments. *P < 0.05 compared with TGF*β*2 (−) (0 ng/mL). ^#^ *P* < 0.05 compared with the group treated with 10 ng/mL TGF*β*2.

**Figure 2:**
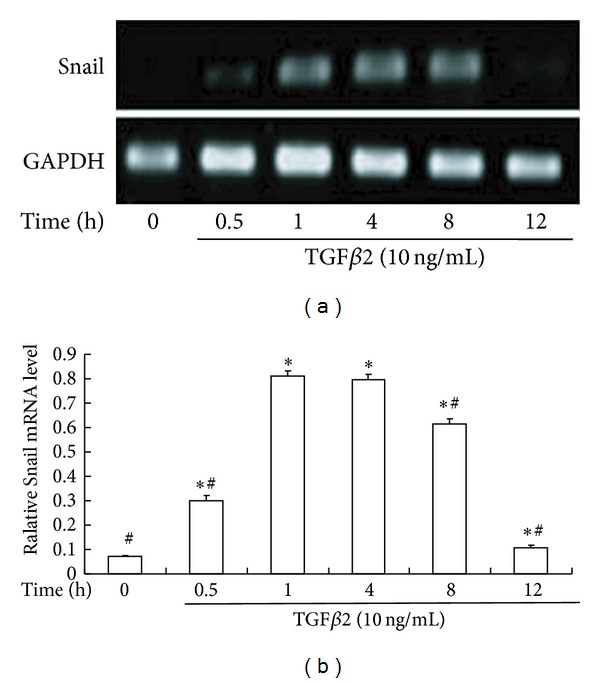
The time course of TGF*β*2-induced expression of Snail mRNA. (a) Representative agarose gel electrophoresis images revealed TGF*β*2-induced early expression of Snail. (b) The summary of triplicated experiments. *P < 0.05 compared with TGF*β*2 (−) (0 h). ^#^ *P* < 0.05 compared with TGF*β*2 (+) (1 h).

To determine whether the expression of *Snail* is regulated by TGF*β*2, we examined the expression and intracellular localization of *Snail* in HLEB3 cells. RT-PCR results indicated that, in the absence of TGF*β*2, there was no *Snail* expression in HLEB3 cells whereas the level of *Snail* mRNA was significantly elevated in cells stimulated with TGF*β*2. TGF*β*2-induced Snail expression was does dependent, and the expression was detected as early as 0.5 h after the treatment (Figures 1 and 2).

**Figure 3:**
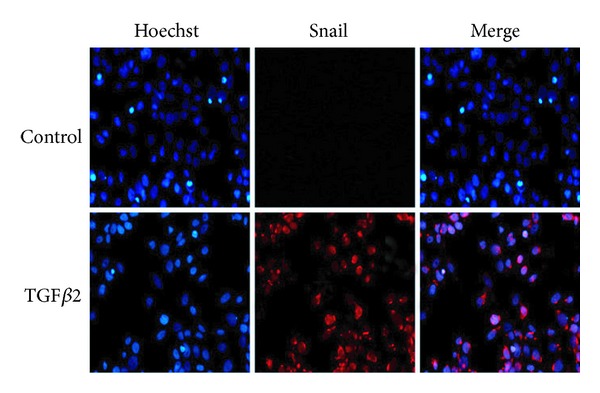
TGF*β*2-induced expression of Snail protein. HLEB3 cells were incubated in the absence or presence of 10 ng/mL TGF*β*2. After 8 hours of culture, cells were immunofluorescence stained with anti-Snail antibody (red) and counterstained with Hoechst (blue). Snail were expressed after TGF*β*2 treatment and located in nuclear (400x).

Consistent with the mRNA expression, Snail protein synthesis was induced after stimulation by TGF*β*2. In the absence of TGF*β*2, the cells showed no immunoreactivity for the protein. However, Snail protein production was greatly increased in the presence of TGF*β*2, and immunostaining was detected mainly in the nucleus and nearby cytosol (Figure 3).

### 3.2. Efficiency of siRNAs Inhibition of *Snail* Expression

**Figure 4:**
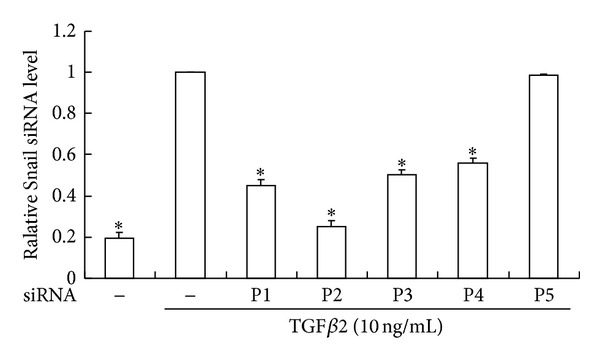
Efficiency of four siRNAs (P1–P4) on Snail expression. Serum starved HLEB3 cells were transfected with human Snail siRNAs (P1–P4) and negative control (P5) before being stimulated with TGF*β*2 for 1 hour. Snail expressions were significantly decreased with the siRNA treatment. The data were collected from 3 experiments. *P < 0.05 compared with siRNA (−)/TGF*β*2 (+) (10 ng/mL).

Four *Snail* siRNAs (P1–P4) inhibited the expression of *Snail* mRNA expression after TGF*β*2 treatment by 55.00% (P1), 74.85% (P2), 49.85% (P3), and 43.98% (P4), respectively (*P* < 0.05), while the negative control siRNA (P5) showed no effects (Figure 4). Because P2 was the most efficient in the inhibition, it was used in the following experiments. 

### 3.3. Role of *Snail* in TGF*β*2-Induced EMT of HLEB3

**Figure 5:**
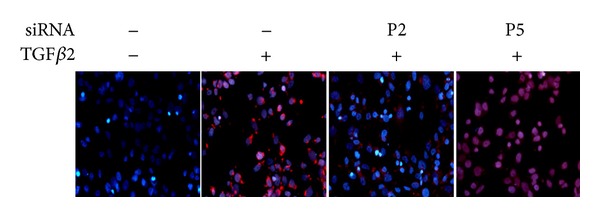
siRNA inhibition of Snail protein expression. Serum starved HLEB3 cells were transfected with human Snail siRNA (P2) and negative control (P5) before being stimulated with TGF*β*2 for 8 hours. Cells were stained with anti-Snail antibody (red) and counterstained with Hoechst (blue). Images were taken by fluorescence microscope (400x).

**Figure 6:**
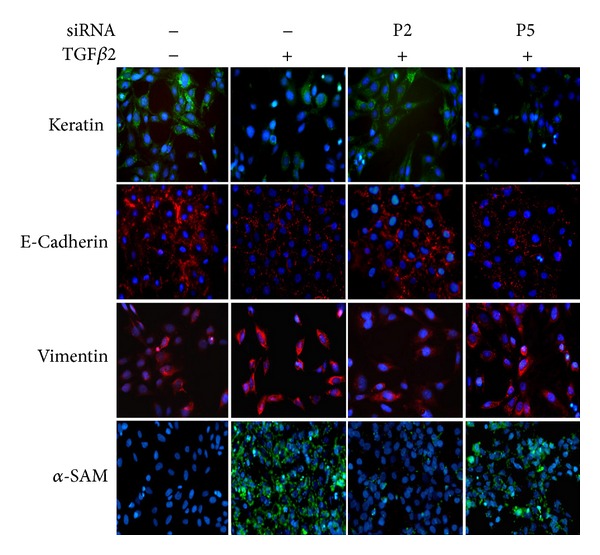
siRNA inhibition of EMT relevant molecules. Serum starved HLEB3 cells were transfected with human Snail siRNA (P2), negative control siRNA (P5). Then cells were stimulated with TGF*β*2 for 24 hours. Various cellular proteins were detected by immunofluorescence staining. Images were taken by fluorescence microscope (400x).

The *Snail *siRNA (P2) reduced the Snail protein expression as well as the mRNA level induced by TGF*β*2 (Figure 5). Although LECs are derived from surface ectoderm, they express vimentin [38] as well as the epithelial surface marker, keratin, and E-cadherin. The vimentin is expressed physiologically in an appropriate amount while overexpression is an evidence of EMT. Immunofluorescence analysis for EMT relevant proteins revealed that keratin, E-cadherin, and vimentin were expressed in normal HLEB3 cells but not *α*-SMA. The TGF*β*2-induced repression of keratin and E-cadherin production was significantly abolished by the *Snail* targeting siRNA. The increase of vimentin and *α*-SMA by TGF*β*2 was inhibited by the siRNA treatment (Figure 6).

**Figure 7:**
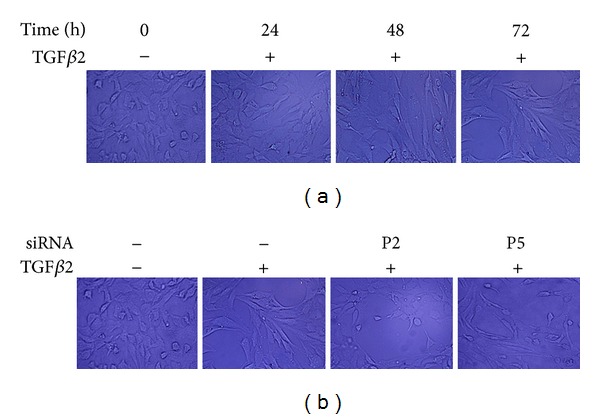
Morphological changes of HLEB3 cells. Serum starved HLEB3 cells were transfected with human Snail siRNA (P2) and negative control siRNA (P5) before the cells were stimulated with TGF*β*2. The morphology of the cells was observed under inverted microscope. (a) TGF*β*2-induced cells became spindly shaped. (b) Snail targeting siRNA prevented the cells from the TGF*β*2-induced morphological change (200x).

The observation of the morphology of HLEB3 cells showed that untreated HLEB3 cells were polygonal with tight junction. After the stimulation of TGF*β*2, the cells became longer and slimmer, spindly shaped as fibroblast, and the junctions of cell-cell were lost. *Snail* targeting siRNA reversed those morphological changes (Figure 7).

**Figure 8:**
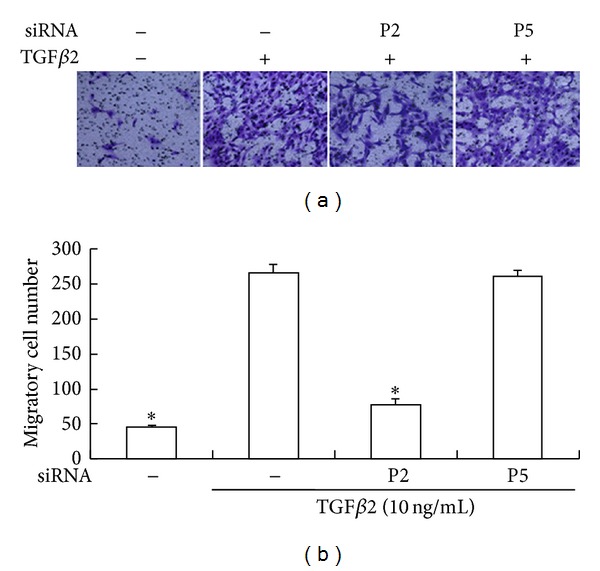
Inhibition of migration ability by Snail siRNA. Serum starved HLEB3 cells were transfected with human Snail siRNA (P2), negative control siRNA (P5) before the cells were stimulated with TGF*β*2 for 48 h. Transwell assay was used to detect the migration ability of cells. (a) Crystal violet stained transmembrane cells under light microscope (100x). (b) The count of migrated HLEB3 cells from triplicated experiments.*P < 0.05 compared with siRNA (−)/TGF*β*2 (+) (10 ng/mL).

There were few untreated HLEB3 cells that passed through the polycarbonate. The migration of TGF*β*2-treated cells was markedly higher than the untreated cells (*P* < 0.05). The treatment of Snail siRNA (P2) significantly blocked the increased migration stimulated by TGF*β*2 (*P* < 0.05) (Figure 8).

## 4. Discussion

In this study, we successfully established a human LEC EMT model and found that *Snail* targeting siRNA can efficiently inhibit TGF*β*2-induced EMT of human LECs, which has not been reported previously. The data indicated the potential to use siRNA approach to suppress development of PCO after cataract surgery.

At present, surgery is the only effective treatment of cataract to restore impaired vision. Unfortunately, many patients suffer a secondary loss of vision over time because of PCO. PCO is the most common long-term complication of cataract surgery. The incidence of PCO is approximately 50% in adults and 100% in children [39–42]. It usually causes a decrease in visual acuity by blocking the visual axis and striae or folds in the posterior capsule. In addition, traction-induced intraocular lens (IOL) malposition, which needed further corrective surgery, can occur during PCO. 

PCO is usually caused by the proliferation, migration, EMT, collagen deposition, and lens fiber regeneration of residual LECs [43–46] because the surgery induces a wound-healing response in the lens. Usually, proliferation of the remaining LECs starts within a few hours after cataract surgery [47]. Proliferation and migration of LECs may precede EMT, and the two events are thought to be independently regulated [48, 49]. Therefore, postsurgical medical inhibition of LECs' proliferation, migration, and EMT would be an option for preventing PCO. 

Myofibroblasts play a central role in the process of tissue fibrosis and scarring. This cell type is derived from both activated fibroblasts and epithelial cells including LECs. Expression of *α*-SMA, a marker for fibroblast-myofibroblast conversion, is mediated by Smads [50]. The transdifferentiation in which an epithelial cell changes its phenotype to a myofibroblast involves many transcription factors including *ZEB (Sip1/dEF1)*, *bHLH (E47/Twist)*, and *Snail*1/2 [51–54]. These transcription factors are upregulated by TGF*β* and directly suppress E-cadherin promoter, which is essential in the maintenance of epithelial phenotype. Expression of *Snail*, the master transcription factor involved in an early step of the EMT, is considered as an important factor in the tissue fibrosis in the eye [7].

We focus on *Snail* because of its relation in cellular proliferation and differentiation. *Snail* is a member of a family of zinc finger-containing transcriptional repressors. *Snail* family is implicated in the transcriptional repression of E-cadherin by interacting with the E-box sequence in the proximal E-cadherin promoter. So, the function of the gene is associated with suppression of the epithelial phenotype [55]. The gene had been shown to be a master gene for early stage of EMT [51, 56, 57]. 

Cho et al. had reported that TGF*β* induced *Snail* expression in mouse lens epithelial cells [36]. It is also reported that *Slug *(*Snail2*, another member of *Snail* superfamily) was expressed in anterior polar cataracts and human lens epithelial cell lines [58]. 

RNA interference has become a standard method for *in vitro* knockdown of any target gene of interest. siRNA can incorporate into a protein complex that recognizes and cleaves target mRNA [59]. Compared to small chemicals for the purpose of inhibition, siRNA mimics RNAi that is a common phenomenon in living creature and is believed to be safe and efficient in the inhibition of a specific gene expression. Four siRNAs against *Snail* were used to avoid off-target effects. Our data suggested that all the designed siRNAs inhibited the expression of *Snail* notably. 

In this study, we have demonstrated that *Snail* is an early responder of TGF*β* in EMT of human LECs. TGF*β*2-treated HLEB3 cells lose their epithelium character and gain mesenchymal feature. *Snails *are implicated in the repression by interacting with the E-box sequence in the proximal E-cadherin promoter, which is associated with morphologic changes in cells that occur during EMT in embryonic development and in tumor cell invasion [27, 34, 35]. We confirmed the similar mechanism in HLEB3 cells. TGF*β*2 changed the polygonal LECs to elongated shape and lost contact with their neighbors. These cells gained notable migration ability. We presumed that the loss of cells' junction is caused by *Snail*-induced E-cadherin's reduction and the contractive property of *α*-SMA contributes to the migration. We found that all these EMT relevant changes were blocked by targeting *Snail*.

In conclusion, our data indicated that TGF*β*2 induces *Snail* expression and EMT of human LECs, and *Snail* is an essential factor in this process. *Snail* targeting siRNA inhibits *Snail* expression and EMT in human LECs, and might be a candidate strategy to prevent subcapsular cataract including PCO. 

## Figures and Tables

**Table 1:** siRNA sequences for snail targeting and negative control.

siRNA duplex	siRNA duplex sequences (5′-3′)	
P1	Sense:	GAAUGUCCCUGCUCCACAAGCdTdT
	Antisense:	GCUUGUGGAGCAGGGACAUUCdTdT
P2	Sense:	GCGAGCUGCAGGACUCUAAUCdTdT
	Antisense:	GAUUAGAGUCCUGCAGCUCGCdTdT
P3	Sense:	CCUUCGUCCUUCUCCUCUACUdTdT
	Antisense:	AGUAGAGGAGAAGGACGAAGGdTdT
P4	Sense:	CAGAUGUCAAGAAGUACCAGUdTdT
	Antisense:	ACUGGUACUUCUUGACAUCUGdTdT
P5	Sense:	UUCUCCGAACGUGUCACGUdTdT
	Antisense:	ACGUGACACGUUCGGAGAAdTdT

Four siRNAs (P1–P4) were designed from the coding sequence of the human Snail gene. The siRNA duplex sequences are listed. A nonspecific, scrambled siRNA duplex as negative control (P5) was used as a control.
